# Les stomies digestives: quel impact professionnel?

**DOI:** 10.11604/pamj.2021.38.118.10700

**Published:** 2021-02-03

**Authors:** Rached Bayar, Seifeddine Baccouche, Zeineb Mzoughi, Abdelkoddous Chelbi, Nafaa Arfa, Lassad Gharbi, Hafedh Mestiri, Mohamed Taher Khalfallah

**Affiliations:** 1 Université de Tunis El Manar, Faculté de Médecine de Tunis, 1007, Tunis, Tunisie,; 2 Service de Chirurgie Viscérale, CHU Mongi Slim, Sidi Daoud, La Marsa, Tunisie

**Keywords:** Stomie, professionnel, SF-36, Stoma, professional, SF-36

## Abstract

**Introduction:**

les stomies digestives constituent l´aboutissement d´un certain nombre d´interventions chirurgicales. Elles peuvent être temporaires ou définitives. Le patient porteur de stomie se trouve confronté à des aléas d´ordre physique et psychologique. Il nécessite donc une adaptation aux changements et une acceptation de la situation qu´elle soit temporaire ou définitive. Le but de ce travail est d´évaluer l´impact des stomies digestives sur la qualité de vie des patients et leur retentissement professionnel.

**Méthodes:**

il s´agit d´une étude rétrospective, menée de janvier 2010 à décembre 2014. Au cours de cette période 115 patients avaient eu des stomies digestives. Parmi ces patients, soixante étaient en âge d´activité professionnelle, avaient un emploi fixe et avaient repris leurs travail; ils constituent l´effectif de notre étude. Le recueil des données était réalisé par un questionnaire spécifique: le questionnaire généraliste SF-36 réalisé en post opératoire.

**Résultats:**

le score moyen pour la qualité de vie globale pour les patients stomisés était 41. Quarante patients avaient une mauvaise qualité de vie avec un score SF-36 qui était inférieur à 50. Notre étude a démontré que les patients stomisés ont éprouvé des difficultés de fonctionnement dans des situations de travail. Quarante-huit patients décrivaient une gêne directement liée à la stomie lors de l´exercice de leurs activités professionnelles. Les causes évoquées étaient d´ordre physique dans 6 cas et d´ordre psychologique dans 3 cas. Six patients étaient mutés vers des postes plus adaptés à leur situation.

**Conclusion:**

l´objectif de la prise en charge des patients porteurs de stomie digestive doit être la réintégration sociale et professionnelle adéquate. Ceci ne peut se concevoir qu´en impliquant le médecin de travail, le psychologue, le stomathérapeute et les associations dans cette prise en charge.

## Abstract (en)

**Introduction:**

digestive stomas result from a certain number of surgical interventions. They may be temporary or definitive. The patient with stoma has potentially damaging physical and psychological problems. Therefore patient´s adaptation to the changes and acceptance of this temporary or permanent situation is essential. This study aims to assess the impact of intestinal stomas on patients´ quality of life and professional life.

**Methods:**

we conducted a retrospective study from January 2010 to December 2014. During the study period, 115 patients had intestinal stomas. Among these patients, sixty were of working age, had a fixed employment and had resumed their work; they made up the study population. Data were collected using a specific questionnaire: the Short Form-36 (SF-36) health questionnaire conducted in the post-operative period.

**Results:**

the average score for the overall quality of life for patients with stoma was 41. Forty patients had poor quality of life with a score SF-36 less than 50. Our study showed that patients with stoma experienced difficulties at work. Forty-eight patients reported discomfort directly linked to stoma during their professional activities. The causes mentioned were physical in 6 cases and psychological in 3 cases. Six patients were moved into more adapted working positions.

**Conclusion:**

the management of patients with digestive stomas should aim to an adequate social and professional reintegration. This can only be done by involving the occupational physician, the psychologist, the stoma therapist and the associations.

## Introduction

Les stomies digestives constituent l´aboutissement d´un certain nombre d´interventions chirurgicales. Elles peuvent être temporaires ou définitives. Elles consistent à aboucher un segment du tube digestif à la peau. Elles sont de plus en plus l´apanage des jeunes patients en âge d´activité professionnelle [1]. Le patient porteur de stomie se trouve confronté à des aléas d´ordre physique et psychologique. Il subit une grande modification de son image corporelle avec des répercussions sur sa vie familiale, sociale et professionnelle [2-4]. Il nécessite donc une adaptation aux changements et une acceptation de la situation qu´elle soit temporaire ou définitive [5,6]. A l´heure actuelle il n´existe pas beaucoup d´études qui s´intéressent à évaluer le retentissement des stomies sur la vie professionnelle des patients [5]. Le but de ce travail est d´évaluer l´impact des stomies digestives sur la qualité de vie des patients et leur retentissement professionnel.

## Méthodes

Il s´agit d´une étude rétrospective, menée de janvier 2010 à décembre 2014. Au cours de cette période, 115 patients avaient eu des stomies digestives. Parmi ces patients soixante étaient en âge d´activité professionnelle, avaient un emploi fixe et avaient repris leurs travail; ils constituent l´effectif de notre étude.

**Les critères d´inclusion:** tous les patients ayant eu une stomie digestive, ayant une activité professionnelle avant l´intervention et ayant repris leur travail après l´intervention.

**Les critères de non inclusion:** les patients retraités ou n´ayant pas d´activité professionnelle fixe et régulière avant l´intervention.

**Les critères d´exclusion:** les patients n´ayant pas repris leur travail après l´intervention.

**Les critères de jugement étaient:** la qualité de vie dans sa globalité, l´état de santé physique et moral des patients porteurs de stomies digestives, le retentissement de la stomie sur l´activité professionnel et le rendement professionnel. Ce dernier critère était évalué d´une façon subjective en laissant le patient apprécier lui-même son rendement.

Le recueil des données était réalisé par un questionnaire spécifique et relatif au retentissement du port de stomie sur l´activité professionnelle; que nous avons élaboré et transcrit dans un canevas standardisé. La qualité de vie ainsi que l´état de santé physique et mental étaient appréciés par le questionnaire généraliste SF-36 réalisé en post opératoire. Il s´agit d´un questionnaire objectif qui a été validé par plusieurs études pour apprécier la qualité de vie chez toute personne ayant un problème de santé notamment après une intervention médicale: c´est une échelle de qualité de vie générique qui explore la santé physique, émotionnelle et sociale. Le SF-36 évalue 8 dimensions de la santé. Pour chacune, on obtient un score variant de 0 à 100, les scores tendant vers 100 indiquant une meilleure qualité de vie. A partir de ces huit échelles, il est possible de calculer deux scores synthétiques qui ont été identifiés par analyse factorielle: un score agrégé de santé physique et un score agrégé de santé mentale.

Le SF-36 se prête aux enquêtes en population générale et peut être administré à des personnes de plus de 14 ans. Un score strictement inférieur à 50 indique une mauvaise qualité de vie. Nous avons complété par un autre questionnaire relatif à l´exercice de leurs professions et à l´évaluation de la prise en charge des patients par le personnel médical et paramédical lors des visites à l´hôpital, chez eux et sur les lieux de travail. Aucun patient n´avait refusé de répondre à nos questions. Les entretiens se sont déroulés aux consultations externes et par téléphone pour les patients qui ne se sont pas présentés aux consultations.

Toutes les données étaient analysées au moyen du logiciel SPSS version 19.0. Nous avions calculé des fréquences absolues et des fréquences relatives (pourcentages) pour les variables qualitatives. Nous avions calculé des moyennes, des médianes et des écarts-types et déterminé les valeurs extrêmes pour les variables quantitatives. Les comparaisons de 2 moyennes sur séries indépendantes étaient effectuées au moyen du test t de Student pour séries indépendantes. Les comparaisons de pourcentages sur séries indépendantes étaient effectuées par le test du khi-deux de Pearson (p=0,05), et en cas de significativité au test du khi-deux et de non-validité de ce test et de comparaison de 2 pourcentages, par le test exact bilatéral de Fisher.

**Consentement:** ce travail ne pose pas de problème éthique. L´accord du comité d´éthique de l´hôpital a été obtenu. Le consentement écrit des patients. Un code a été attribué à chaque questionnaire, donc à chaque patient pour garantir la confidentialité des données.

## Résultats

Durant la période d´étude, 115 patients avaient nécessité la confection d´une stomie digestive. Soixante patients avaient repris leur travail. Ils avaient un emploi fixe avec une activité professionnelle jugée normale avant l´intervention. Ces patients constituent l´effectif de notre étude.

**Etude de la population:** la médiane d´âge des patients était de 46 ans avec des extrêmes allant de 20 à 54 ans. Il s´agissait de 38 hommes et 22 femmes avec un sex-ratio de 1,72. Une iléostomie était réalisée chez 34 patients. Elle était temporaire chez 28 patients et définitive chez 6 patients. Une colostomie était réalisée chez 26 patients. Elle était temporaire chez 6 patients et définitive chez 20 patients. La pathologie ayant nécessité la réalisation de stomies digestives était cancéreuse dans 26 cas, une maladie inflammatoire chronique de l´intestin dans 12 cas, une occlusion intestinale aiguë dans 10 cas, une péritonite aiguë dans 6 cas et une ischémie mésentérique dans 6 autres cas (Tableau 1).

**Tableau 1:** type de stomie en fonction de la pathologie

	Cancer colorectal	Colite inflammatoire	Occlusion intestinale	Péritonite aiguë	Ischémie mésentérique	
Iléostomie-iléo-colostomie	14	6	6	4	4	34
Colostomie	12	6	4	2	2	26
Total	26	12	10	6	6	60

**Figure 1:**
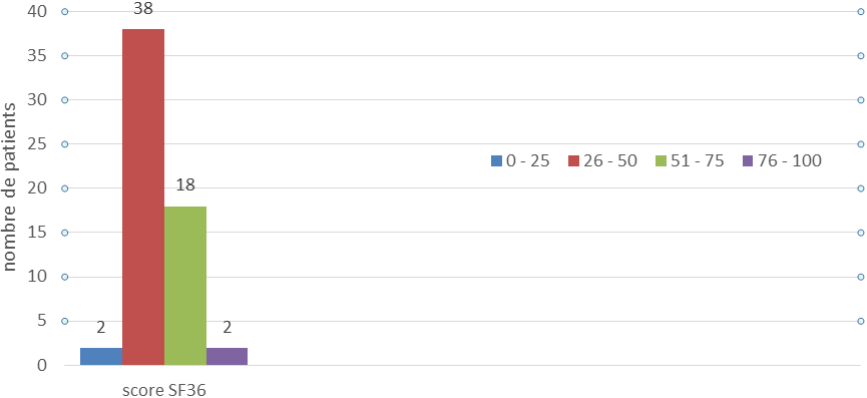
nombre de patients en fonction du score SF-36

**Figure 2:**
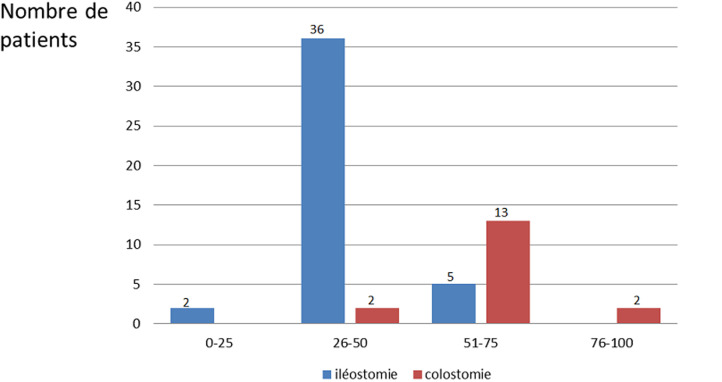
score SF-36 chez les patients porteurs d'iléostomie ou colostomie

**Etude de la qualité de vie:** quarante patients avaient une mauvaise qualité de vie avec un score SF-36 qui était inférieur à 50 (Figure 1). La qualité de vie variait en fonction du type de stomie. En effet, parmi les 40 patients ayant une mauvaise qualité de vie, 38 étaient porteurs d´une iléostomie. Pour les 20 patients ayant une qualité de vie jugée moyenne à bonne 15 étaient porteurs d´une colostomie (Figure 2). Les patients porteurs de colostomie avaient une meilleure qualité de vie et la différence entre les deux groupes de patients était significative (p = 0,028).

**Le retentissement professionnel:** le délai moyen de reprise de travail était de 77 jours pour les patients porteurs de colostomie et de 103 jours pour les patients porteurs d´iléostomie et la différence entre les deux groupes était significative (p = 0,032). Quarante-huit patients décrivaient une gêne directement liée à la stomie lors de l´exercice de leurs activités professionnelles. Trente-huit étaient porteurs d´une iléostomie et 10 d´une colostomie. Il s´agissait d´irritation et de prurit chez 18 patients, de décollement fréquent du support chez 12 patients, de douleur au niveau de l´orifice stomial chez 11 patients et de perception de mauvaises odeurs chez 7 patients. Trente patients décrivaient une baisse considérable de leurs rendements professionnels. Parmi ces patients, 26 étaient porteurs d´iléostomie et 4 de colostomie. La différence entre ces deux groupes est significative (p = 0,04). Cette baisse du rendement était en rapport avec un changement fréquent des supports et des sacs de stomies dans 25 cas et de douleur ou de l´irritation dans 5 cas. Parmi notre effectif 9 patients avaient arrêté secondairement de travailler avec un délai moyen de 182 jours. Cet arrêt était imputé directement à la stomie. Les causes évoquées étaient d´ordre physique dans 6 cas et d´ordre psychologique dans 3 cas. Six patients étaient mutés vers des postes plus adaptés à leur situation.

**La qualité de prise en charge des patients:** à domicile, aucun patient n´avait reçu la visite d´un stomathérapeute, la gestion de la stomie était exclusivement réalisée par le patient lui-même (80%) et les membres de sa famille (20%). A l´hôpital; 30% des patients considérait ne pas être assez préparer psychologiquement avant l´intervention au port de la stomie et 83% disaient avoir été mal pris en charge en postopératoire. Sur les lieux de travail; uniquement cinq patients avaient eu une visite médicale par le médecin de travail. Parmi ces patients, 3 affirmaient que lors de cette visite les médecins n´avaient pas posés de questions relatives à la stomie. Deux patients avaient signalé un retentissement psychologique important lors de l´exercice de leurs activités professionnelles et aucun n´avait eu une prise en charge psychologique.

## Discussion

Le port d´une stomie digestive a un impact psychologique et physique très important [2,3]. L´impact physique parait plus important puisque la présence même d´un appareillage peut être gênante quant à la pratique de certaines professions [4]. Notre étude a montré que 2/3 des patients porteurs de stomies digestives avaient une mauvaise qualité de vie avec un score SF-36 inférieur à 50. Ce taux est plus élevé que la majorité des résultats rapportés dans la littérature. Cette différence de résultat s´explique d´une part par l´utilisation de plusieurs scores différents pour évaluer la qualité de vie et d´autre part par l´âge relativement jeune de nos patients. En effet, il a été démontré que les jeunes patients atteints de maladie inflammatoire de l´intestin acceptaient mal le port de stomie même temporaire et de ce fait avaient une mauvaise qualité de vie [7]. Les patients plus âgés ou atteints de cancers traités ou en cours de traitement acceptaient leur situation et avaient une qualité de vie meilleur [7]. L´étude de deux sous-groupes de patients porteurs: d´iléostomie et de colostomie avait montré que l´iléostomie était plus pourvoyeuse d´une baisse de la qualité de vie que la colostomie. Le délai moyen de la reprise du travail était plus précoce pour les patients porteurs de colostomie que pour les patients porteurs d´iléostomie. L´iléostomie était plus associée à une gêne d´ordre physique en milieu professionnel et une baisse du rendement que la colostomie.

L´iléostomie est surtout gênante de par son débit important et l´irritation cutanée provoquée par le liquide digestif, ce qui rend le changement de sac handicapant, pluriquotidien surtout lorsqu´il est réalisé en milieu professionnel. Il s´y ajoute les troubles de sommeil dus à la vidange du sac la nuit ainsi que les douleurs sont les principaux facteurs qui influence le rendement professionnel [4-7]. En fait, parmi les facteurs influençant la reprise de travail, on trouve en premier lieu le défaut d´appareillage et l´irritation cutanée. Ces complications sont spécifiques de l´iléostomie ce qui explique le faible taux de reprise du travail après iléostomie. La non-administration systématique de ralentisseurs de transit semble aggraver davantage cette gêne [6]. Pour les colostomies, l´odeur reste le facteur limitant le plus important associé au décollement du sac vu le poids du contenu, mais son impact reste moins important que la gêne causée par les iléostomies [2].

L´impact psychologique est difficile à évaluer. Il est secondaire à la modification de l´image de soi et du regard de l´autre. Il existe certainement une susceptibilité individuelle et variable d´une personne à une autre [6-8]. Actuellement il est admis que les thérapies de groupe et la prise en charge spécialisé impliquant stomathérapeute et psychologue entraine une meilleure acceptation de l´image de soi et par la même une amélioration de la qualité de vie et une meilleure réhabilitation professionnelle [1]. Un arrêt secondaire de l´activité professionnelle était noté chez 9 patients et 5 patients avaient eu une mutation. L´arrêt du travail est le résultat de plusieurs facteurs: la gêne, la baisse du rendement et l´absence de prise en charge psychologique. En effet les patients porteurs de stomie ont une tendance manifeste à l´isolement et la réinsertion professionnelle est souvent difficile [6]. Mais les causes exactes expliquant l´arrêt du travail restent à déterminer. Ceci peut se faire par des questionnaires plus adaptés à l´évaluation de l´impact des stomies sur l´activité professionnelle.

La qualité globale de la prise en charge aussi bien dans les structures sanitaires, à domicile et sur les lieux de travail était très mauvaise. Ceci s´explique par l´absence de formation aussi bien des médecins que du personnel paramédical dans la prise en charge des patients en périopératoire. En effet, le personnel médical et paramédical en Tunisie ne dispose pas de formation spécialisée dans la prise en charge des patients porteurs de stomie. Il n´existe pas de statut de stomathérapeute reconnu. La prise en charge des patients par un personnel qualifié améliore nettement la qualité de vie [1].

## Conclusion

L´objectif de la prise en charge des patients porteurs de stomie digestive doit être la réintégration sociale et professionnelle adéquate. En effet, ces patients ont besoin en permanence d´un accompagnement et d´un soutien afin de répondre à leurs besoins en termes d´information et d´éducation. Ceci ne peut se concevoir qu´en impliquant le médecin de travail, le psychologue, le stomathérapeute et les associations dans cette prise en charge.

### Etat des connaissances sur le sujet

- Les stomies ont un retentissement physique et psychologique;
- Les stomies ont un retentissement social et a priori professionnel.

### Contribution de notre étude à la connaissance

- L´évaluation objective du retentissement professionnel par un questionnaire a été fait;
- Une comparaison du retentissement professionnel de la colostomie et de l´iléostomie est présentée.
